# Prevalence and molecular characterization of *Clostridium difficile *isolated from feedlot beef cattle upon arrival and mid-feeding period

**DOI:** 10.1186/1746-6148-8-38

**Published:** 2012-03-28

**Authors:** Marcio C Costa, Richard Reid-Smith, Sheryl Gow, Sherry J Hannon, Calvin Booker, Joyce Rousseau, Katharine M Benedict, Paul S Morley, J Scott Weese

**Affiliations:** 1 Department of Pathobiology, Ontario Veterinary College, University of Guelph, Guelph, Canada; 2 Public Health Agency of Canada, Guelph and Saskatoon, Guelph, Canada; 3 Feedlot Health Management Services Ltd., Okotoks, Canada; 4 Department of Clinical Sciences, Colorado State University, Fort Collins, CO, USA

**Keywords:** Public health, Foodborne, Oxytetracycline, Antimicrobials

## Abstract

**Background:**

The presence of indistinguishable strains of *Clostridium difficile *in humans, food animals and food, as well as the apparent emergence of the food-animal-associated ribotype 078/toxinotype V as a cause of community-associated *C. difficile *infection have created concerns about the potential for foodborne infection. While studies have reported *C. difficile *in calves, studies of cattle closer to the age of harvest are required. Four commercial feedlots in Alberta (Canada) were enrolled for this study. Fecal samples were collected at the time of arrival and after acclimation (< 62, 62-71 or > 71 days on feed). Selective culture for *Clostridium difficile *was performed, and isolates were characterized by ribotyping and pulsed-field gel electrophoresis. A logistic regression model was built to investigate the effect of exposure to antimicrobial drugs on the presence of *C. difficile*.

**Results:**

*Clostridium difficile *was isolated from 18 of 539 animals at the time of feedlot arrival (CI = 2.3-6.1) and from 18 of 335 cattle at mid-feeding period (CI = 2.9-13.1). Overall, there was no significant difference in the prevalence of *C. difficile *shedding on arrival versus mid-feeding period (*P *= 0.47). No association between shedding of the bacterium and antimicrobial administration was found (*P *= 0.33). All the isolates recovered were ribotype 078, a toxinotype V strain with genes encoding toxins A, B and CDT. In addition, all strains were classified as NAP7 by pulsed field gel electrophoresis (PFGE) and had the characteristic 39 base pairs deletion and upstream truncating mutation on the *tcd*C gene.

**Conclusions:**

It is apparent that *C. difficile *is carried in the intestinal tracts of a small percentage of feedlot cattle arriving and later in the feeding period and that ribotype 078/NAP7 is the dominant strain in these animals. Herd management practices associated with *C. difficile *shedding were not identified, however further studies of the potential role of antimicrobials on *C. difficile *acquisition and shedding are required.

## Background

*Clostridium difficile *is a Gram-positive, spore-forming bacterium responsible for *C. difficile *infection (CDI) in humans [1], a serious and increasingly problematic disease. A remarkable change in the epidemiology of CDI has been encountered over the past 10 years, with increasing incidence, mortality and relapse rates in humans [2,3]. Additionally, while classically a hospital-associated pathogen predominantly affecting elderly individuals, there are increasing reports of community-associated CDI, including disease in younger individuals and people with few or no traditional risk factors [1,4,5].

The source of infection for community-associated cases of CDI remains uncertain, however foodborne infection has been suggested [4]. Indeed, several studies have recovered spores of *C. difficile *from food products including retail meat [6-9], yet the source of contamination for food products has not been identified. Contamination of carcasses during slaughter and processing is most likely [10,11], but the presence of dormant spores of the bacterium in the muscle tissues of food animals should also be considered [12].

While currently unproven, concerns about zoonotic and foodborne transmission of CDI are reasonable considering reports of isolation of *C. difficile *from animals. Concern has been heightened by the apparent increase in CDI in humans caused by the *C. difficile *ribotype 078 [4,13,14], a strain that has been associated with community-associated infection [15,16] since this is the dominant *C. difficile *strain among food animals [15,17-19] and has also been recovered from meat products [8,9].

High prevalences of *C. difficile *shedding have been reported in food animals, yet most studies have involved young animals well before the time of slaughter. Studies of animals performed nearer to the time of slaughter are likely more relevant for assessment of the potential for foodborne exposure, yet limited epidemiological investigations have been performed in feedlot cattle [11,20].

The objectives of this study were to evaluate the shedding of *C. difficile *by adult feedlot cattle upon arrival and after acclimation to the feedlot diet and environment, and to characterize the recovered isolates. In addition, the potential association between the use of antimicrobial drugs and shedding of the bacterium was also investigated.

## Results

A total of 539 cattle were sampled from 4 feedlots, ranging from 121 to 179 cattle per feedlot (mean 135). Adjusting for lack of independence in pens, *C. difficile *was isolated overall from 3.7% of 539 (CI = 2.3-6.1) animals at the time of feedlot arrival in 34 unique pens and from 6.2% of 335 (CI = 2.9-13.1) cattle at mid-feeding period in 22 unique pens. No individuals were positive at both sampling times.

Results of the regression analysis using generalized estimating equation (GEE), accounting for the date of sampling during the feeding period (< 62 days on feed (DOF), 62-71 DOF, or > 71DOF) and for the effects of repeated sampling are presented in Table 1. There was no statistically detectable difference in the likelihood of recovery of *C. difficile *based on DOF (*P *= 0.47).

**Table 1:** Univariable associations of use of antimicrobials with prevalence of *C.difficile *and prevalence at different periods*

Class of antimicrobial	Odds ratio	95% CI	*P*-value
IN FEED			0.33

Tetracycline	0.77	0.55-1.08	

DAYS ON FEED			0.47

< 62	4.25	0.73-24.63	

62-71	1.07	0.21-5.38	

> 71	Referent	Referent	

* controlling for lack of independence sampling

Sixty-four individuals were treated with parenteral antimicrobials during the study period including beta-lactams (2), macrolides (32), phenicols (1), quinolones (1), sulfonamides (1) and tetracyclines (27). The sum of the Animal Defined Daily Dose (ADD), which quantifies the total amount of antimicrobials administered for each period and for the total risk period were 11, 96, 3, 3, 3 and 54 for each of the drug classes, respectively. Tetracycline was the only in-feed antimicrobial used and was given to all individuals, with a summed ADD of 1278.4. As shown in Table 1, there was no significant association between the use of tetracycline and the prevalence of *C. difficile *in these animals (*P *= 0.33). Results for models regarding the remaining antimicrobials are not shown, as the univariable logistic regression models did not converge.

**Figure 1:**
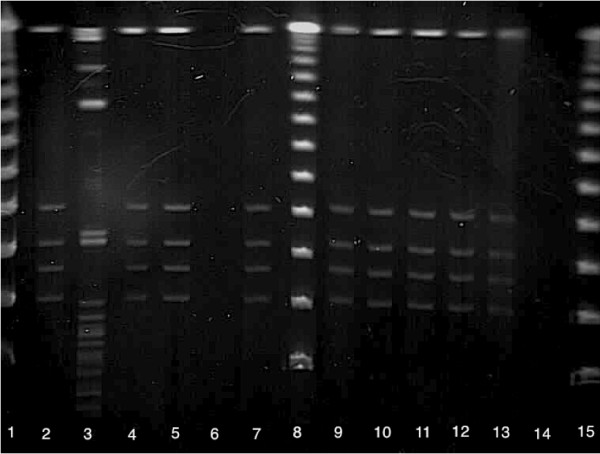
**Pulsed-field gel electrophoresis of *Clostridium difficile *isolates recovered from beef cattle entering feedlots**. Pulsed-field gel electrophoresis of *Clostridium difficile *isolates recovered from beef cattle entering feedlots. **Figure 1 legend: **Lanes 1, 8 and 15 contain a PFGE ladder (50-1000 kb). Lane 3 has an unrelated sample and lanes 6 and 14 are empty. The remaining lanes show NAP7 *C. difficile*.

One isolate could not be recovered after storage and therefore, molecular analysis was performed on the remaining 35 isolates. All the isolates recovered were classified as ribotype 078 and were positive for the toxin producing genes *tcd*A*, tcd*B and *cdt*A. All isolates were indistinguishable on PFGE and classified as North American pulsotype 7 (NAP 7) (Figure 1). As expected, sequencing of the *tcd*C gene identified a 39 bp deletion and C184T upstream truncating mutation, consistent with the *tcd*C-A genotype according to Curry et al. [21].

## Discussion

The prevalence of feedlot cattle shedding *C. difficile *was low at both sampling times. There is a limited number of studies concerning the epidemiology of *C. difficile *in the bovine species and the data available in the literature are largely restricted to calves [17,18,22-24]. The low shedding prevalence of *C. difficile *in cattle arriving at feedlots reported here is in agreement with the prevalence reported by Indra et al. [2] in cows (4.5%) and slightly higher than the one reported by Rodriguez-Palacios et al. [20] in feedlot cattle at harvest age (1.8%). However, our results indicate a lower shedding prevalence than the one found by Rodriguez-Palacios et al. [11] whom isolated the bacterium from 12.9% of animals arriving to feedlot. The reasons for this difference cannot be explained, however stressing from shipping and different management conditions prior to arrival such as hygiene, population, stocking density, animal mixing and antimicrobial administration should be considered, along with potential geographic variation and differences in methodology.

A high prevalence of *C. difficile *shedding has been reported in young calves. The bacterium was recovered from 14.9% of healthy calves in one study [24] and from 12.7% in another [18], but it can be as high as 51% in veal calves [17]. In addition, *C. difficile *has been detected by PCR in feces of 33.8% of calves submitted for post-mortem examination [23]. The lower prevalence in this and the above-cited studies of older animals is perhaps not surprising given increasing evidence of a strong effect of age on *C. difficile *shedding prevalence, something that has been noted in swine [25-27] and horses, [28], as well as humans [29]. Costa et al. [17] also observed a significant decrease in shedding prevalence (from 32% to 2%) as veal calves became older, which is in agreement with Hoffer et al. [22], who isolated *C. difficile *from only one out of 204 veal calves sampled at slaughter. The reasons for a higher shedding prevalence in young individuals are uncertain but competition from a more adapted intestinal microflora in older animals may make colonization more difficult. In addition, stress from birth adaptation, a naïve immune response, the type of feeding and the different management systems could also explain the increased prevalence in younger animals.

The potential for antimicrobial drugs to disrupt the normal intestinal microflora leading to *C. difficile *overgrowth has been shown in other species, including humans [30] and horses [28]. Therefore, in order to further investigate the effect of *C. difficile *shedding by animals from feedlot, antimicrobial exposure of cattle was investigated. Antimicrobial exposure, particularly tetracyclines, was common however no influence on *C. difficile *shedding was identified. The lack of an identifiable association between the use of antimicrobials and shedding of *C. difficile *reported here agrees with the findings of Rodriguez-Palacios et al. [11].

Ribotype 078, the only strain found here, can cause CDI in humans [4,14] and has been increasingly associated with community-associated CDI [13,15]. A study of human and animal ribotype 078 isolates using highly discriminatory multilocus variable number tandem repeat analyses indicated they were closely related, suggesting that the bacterium may be transmitted between humans and animals [31]. The predominance of this strain was not particularly surprising since it is typically associated with food animals, has been found in various studies of pigs and cattle and it is the most commonly reported strain in pigs in Canada [2,17-19,22,31,32]. In addition, the strain has been prevalent (between 73% and 86%) among isolates recovered from some retail beef samples [8,9]. Another interesting aspect was the finding that no cattle were positive at both sampling times. This suggests that persistent carriers are not common, and that *C. difficile *may be transiently shed and circulated throughout animal populations. Inconsistent or transient shedding has also been reported in dogs [33] and horses [34]. Further studies involving more frequent sampling would help elucidate the on-farm cycling of *C. difficile*.

The predominance of clones classified as ribotype 078/NAP7 recovered from beef cattle reported here (100%) reinforces the results of other studies in the bovine species [17-19,22]. However, other researchers found that only 2 out of 24 [11] and 1 out of 17 [20] isolates recovered from cattle were ribotype 078. In addition, it was interesting that other strains, including 027/NAP III, found in retail beef [6,8,9] were not isolated in the present study.

The prevalence of shedding of *C. difficile *ribotype 078 by feedlot cattle reported here is an important information for a better understanding of *C. difficile *epidemiology in adult beef cattle. The low prevalence upon feedlot arrival and later in the feeding period must be considered when interpreting food contamination and foodborne risks. The lack of an effect of antimicrobials exposure was interesting and while a study such as this cannot completely discount a role of antimicrobials in the epidemiology of *C. difficile *in cattle, it is clearly not a major determinant, at least on these farms.

## Conclusions

We conclude that the prevalence of *C. difficile *in adult cattle was low at arrival and at mid-feeding period from feedlots. No association was found between exposure to antimicrobial drugs or between days on feed and the prevalence of *C. difficile *in this collection of samples. Ribotype 078/NAP7 was the only strain present among all isolates recovered. It is apparent that *C difficile *is carried in the intestinal tracts of a small percentage of beef cattle going to slaughter, but its public health significance requires further study.

## Methods

### Animal selection

Cattle enrolled in this study were managed at four western Canadian feedlots in south central Alberta, under production conditions that are typical of those used at large commercial cattle feedlots throughout western Canada and the United States. Feedlots had one-time capacities between 15,000 and 20,000 animals, with pens capable of housing 50 to 350 animals. Animals are housed in open-air, dirt-floor pens arranged side by side with central feed alleys and 20% porosity wood-fence windbreaks. All feedlots have modern cattle handling facilities. Each animal handling facility has a hydraulic chute equipped with an individual animal scale, a chute-side computer with software for individual animal data collection and management (*i*FHM*S*, Feedlot Health Management Services Ltd., Okotoks, Alberta) and separation alleys to facilitate the return of animals to designated pens. All animal handling and sampling procedures were approved prior to the initiation of the study by the University of Guelph Animal Care Committee, the Animal Care Committee of Feedlot Health Management Services (FHMS) and the Institutional Animal Care and Use Committee of Colorado State University.

Candidate animals utilized in the study were procured through the auction market system across western Canada. Various cattle types were fed at these feedlots including cattle of various entry weights, age classes (calves and yearlings), frame sizes, sources (e.g., ranch-direct cattle and back-grounded cattle), and genders (bulls, steers and heifers). A 2-stage random sampling plan was used to determine which pens and animals within those pens were selected for enrolment. Animals were allocated to the study from January 17, 2009 to September 11, 2009. During the enrolment period, 30% of all new pens of cattle were randomly selected for inclusion in the study using a pen randomization table as the cattle arrived at the feedlot. Within each selected pen, 10% of all animals in that pen were then randomly enrolled in the study at initial processing using an individual animal randomization table. Cattle enrolled in the study weighed a mean of 375 kg (152 to 513 kg).

All animals enrolled in the study were subject to standardized animal health management and feedlot production procedures as per the protocols developed by the feedlot animal health/production consultants (FHMS). In brief, each animal received a unique individual animal identification ear tag, a trial-specific ear tag to help identify individuals for future sample collection, a subcutaneous hormonal growth implant in the middle third of the ear, vaccine(s) to immunize against selected bacteria and viruses that cause disease in feedlot cattle, and application of topical avermectin for internal and external parasite control. In animals at higher risk of developing disease, a parental antimicrobial was administered as part the prevention and control strategies for bovine respiratory disease. Water and standard mixed complete feedlot diets, formulated to meet or exceed the National Research Council nutritional requirements for beef cattle feedlot cattle, were offered *ad libitum *throughout the feeding period.

Individual animals enrolled in the trial were sampled twice over the course of the study: at the time of arrival and initial processing, and then again at various times in the middle of the feeding period when cattle were processed again to perform standard feedlot management procedures. Feces were collected from individual animals per rectum using a new palpation sleeve (#33, Almedic, Montreal, Canada) for collection and transfer (minimum 4 g) into a new sterile plastic fecal cup (# 109117, Globe Scientific, Paramus, New Jersey). Fecal samples were labelled, refrigerated (4°C) and transported to FHMS within 7 days of collection. At FHMS, samples were placed into ZipLoc bags and shipped (once a week in a chilled cooler, by air courier (Purolator Corporation) from Calgary, Alberta to the microbiology laboratory (University of Guelph, Guelph, Ontario) for further processing.

Each fecal sample collected over the course of the trial was assigned a unique identification number to ensure blinding of the laboratory staff and uniform labelling of samples. All treatments of cattle housed in the pens of cattle that were enrolled and sampled for the Individual antimicrobial use data were recorded at each feedlot over the course of the study using a chute-side computer system (*i*FHM*S*, FHMS). These data were available for each animal and included the product, the dose, the route and the number of days administered. Data on both individual animal and in-feed antimicrobial exposure were collected, with the in-feed data compiled from the pen-based feeding records. All study data were subsequently compiled, collated in a computer spreadsheet program (Microsoft Office Excel 2003), and verified.

### Clostridium difficile culture

Approximately 2 g of feces were inoculated into 9 mL of *C. difficile *moxalactam norfloxacin (CDMN) enrichment broth (Oxoid Ltd; Nepean, ON Canada) containing 0.1% sodium taurocholate and incubated anaerobically at 37°C for 7 days. Two millilitres of broth were then added to 2 mL of anhydrous alcohol and incubated at room temperature for 60 min. After centrifugation (3,980 rcf for 10 min), the pellet was inoculated onto CDMN (Oxoid Ltd; Nepean, ON Canada) agar and incubated in an anaerobic chamber at 37°C for 48 h and, if negative, re-checked 3 days later. Isolation and identification of *C. difficile *was based on the characteristic morphology and odour of the colonies, Gram stain and the presence of the L-proline aminopeptidase activity (Remel Inc, Lenexa, KS, USA). One single colony for each isolate was subcultured and stored at -80°C and re-cultured prior to molecular analysis.

### Molecular analysis

*Clostridium difficile *was grown on blood agar for 24 h and approximately 10 colonies were suspended in 1 mL of distilled water and centrifuged at 12,100 rcf for 1 min. The supernatant was discarded and 200 μL of a commercial DNA extraction kit (InstaGene Matrix; Bio-Rad, Richmond, CA, USA) were added and incubated at 56°C for 30 min and at 100°C for 8 min. The mixture was centrifuged and 200 μL of the supernatant were frozen at -20°C until processing.

Ribotyping was performed as described by Bidet et al. [35]. Ribotype patterns were evaluated visually and compared to an internal library of ribotypes. The international numerical designation (e.g., ribotype 078) was used for bacterial strains recognized as a known international ribotype based on comparison with reference strains. A multiplex PCR was used for detection of genes encoding toxin A (*tcdA*) and toxin B (*tcdB*) as described by Lemee et al. [36]. A second PCR was performed for detection of toxin A gene constitutive difference between A-/B+ strains and A+/B+ strains, and thus, identification of toxin A negative strains was performed according to Kato et al. [37]. Detection of *cdt*A, the gene encoding for the enzymatic component of CDT, was performed according to Stubbs et al. [38]. Sequence analysis of the *tcd*C gene was performed [39] and the result was classified according to Curry et al. [21].

Pulsed-field gel electrophoresis was performed following the protocol used by Miller et al. [40] with modifications. Briefly, *C. difficile *was cultured for 48 h and inoculated into 3 mL of pre-reduced brain and heart infusion (BHI) solution and grown anaerobically for 6 h. The solution was then adjusted to have an optical density (OD)_600 _between 0.3 and 0.7. Four hundred microlitres of the solution were centrifuged at 12,000 rcf for 1 min and the pellet suspended in 150 μL of cell lysis buffer and added 150 μL of melted 1% SeaKem Gold PFGE agarose (Cambrex BioScience Rockland Inc., ME, USA) plus 1% SDS for pipetting into plug molds. After solidified, plugs were transferred into 500 μL of lysis buffer added by 25 μL of lysozyme (final concentration 20 mg/mL) and 25 μL of mutanolysin (final concentration 12.5 U/mL) and incubated at 37°C overnight. The solution was then replaced by 500 μL of proteinase K (PK) buffer added by 25 μL of PK (final concentration 20 mg/mL) and incubated overnight at 56°C for 4 h. Plugs were rinsed three times with 1 × TE buffer and placed on a shaker for 5 min. The last step was repeated once and three more washes were performed with intervals of 10, 15 and 20 min. The solution was replaced by 150 μL of buffer A (New England Biolabs, ON, Canada) and after 10 min replaced by buffer A plus 60 U of restriction enzyme *Sma*I and incubated at 25°C overnight. Half of the plug was cut off and transferred to wells of a 1.3% pulsed-field certified agarose gel (BioRad, CA, USA). DNA separation was performed in a CHEF-DR II chamber (BioRad, CA, USA) added of 2.2 L of 0.5× TBE buffer plus 500 μL of 0.2 M thiourea, set to run for 22 h at 6 V/cm with initial switch time of 1 sec and final switch time of 40 sec. The gel was stained in ethidium bromide and images were obtained using a computerized system (SynGene, Synoptics, MD, USA).

### Antimicrobial use data

Individual animal exposure data regarding antimicrobial drugs were recorded at each feedlot over the course of the study using a chute-side computer system (iFHMS, Okotoks, Alberta). These data included the product used, the dose, and the date and route of administration. All study data were subsequently compiled, collated in a computer spreadsheet and verified. Ionophores, and coccidiostats were not included in this analysis.

Dosage information for exposures to antimicrobial drugs was converted into an Animal Defined Daily Dose (ADD). The ADD metric represents the number of days of treatment for an animal based on an assumed average maintenance dosage needed for clinical therapy. Dosage conversion to ADD was based on the expected length of drug effect as indicated by approved dosages.

### Statistical analysis

The least square means estimates and 95% confidence intervals for the prevalence of *C. difficile *at arrival and the second (post-arrival) time point were modelled using logistic regression. Regression analysis using generalized estimating equation (GEE) methods, was used to correct prevalence estimates for lack of independence related to sampling of multiple individuals from the same pens using a compound symmetry (exchangeable) correlation structure. Logistic regression was performed using commercially available software (SAS version 9.2, SAS Institute Inc, Cary, NC).

Date of sampling during the feeding period (< 62 days on feed (DOF), 62-71 DOF, or > 71DOF) and exposure of cattle to antimicrobial drugs. Antimicrobial exposure data were summarized as ADDs administered parenterally or in feed between arrival and second sampling, by class of antimicrobial drug, and by route of administration (beta lactams, macrolides, phenicols, quinolones, tetracyclines, and sulfonamides for parenteral exposures and tetracyclines for in-feed exposures).

The outcome for the logistic models was the presence or absence of *C. difficile*. Variables were screened in univariable models to determine those to be included in multivariable model building using a critical alpha for inclusion of 0.25. Multivariable models were not assessed since none of the antimicrobial exposure variables met the inclusion criteria. Odds ratios, 95% confidence intervals (95%CI), and the associated P-values were reported from logistic regression models.

## Authors' contributions

MC: Molecular analysis of isolates. Analysis and interpretation of data. Writing manuscript. CB and SH: Participated in study design, coordinated sample collection and antimicrobial use data collection, and assisted with interpretation of data analysis. RR-S and SG: Participated in study design, project coordination, and project oversight, and assisted with interpretation of data analysis. JSW: Principal investigator in charge of study design and laboratory procedures. Analysis and interpretation of data. Critical review. JR: Isolation of *C. difficile*. PM and KB: Participated in study design, project coordination and oversight, conducted and interpreted statistical analyses. All authors read and approved the final manuscript.
